# Comprehensive Genomic Profiling in Non-Myeloid Hematologic Malignancies Identifies Variants That Can Alter Clinical Practice

**DOI:** 10.3390/hematolrep16040059

**Published:** 2024-09-30

**Authors:** Chenyu Lin, Katherine I. Zhou, Michelle F. Green, Bennett A. Caughey, John H. Strickler, Michael B. Datto, Matthew S. McKinney

**Affiliations:** 1 Division of Hematologic Malignancies & Cellular Therapy, Department of Medicine, Duke University School of Medicine, Durham, NC 27705, USA; 2 Division of Medical Oncology, Department of Medicine, Duke University School of Medicine, Durham, NC 27705, USA; 3 Labcorp Oncology, Durham, NC 27704, USA; 4 Division of Hematology and Oncology, Department of Medicine, Massachusetts General Hospital, Boston, MA 02114, USA; 5 Department of Pathology, Duke University School of Medicine, Durham, NC 27705, USA

**Keywords:** non-myeloid hematologic malignancies, comprehensive genomic profiling, actionable variants

## Abstract

Background: Comprehensive genomic profiling (CGP) is frequently adopted to direct the clinical care of myeloid neoplasms and solid tumors, but its utility in the care of lymphoid and histiocytic cancers is less well defined. Methods: In this study, we aimed to evaluate the frequency at which mutations identified by CGP altered management in non-myeloid hematologic malignancies. We retrospectively examined the CGP results of 105 samples from 101 patients with non-myeloid hematologic malignancies treated at an academic medical center who had CGP testing between 2014 and 2021. Results: CGP revealed one or more pathogenic or likely pathogenic variant in 92 (88%) of samples and 73 (72%) of tested patients had one or more mutations with diagnostic, prognostic, or therapeutic significance. The identification of a resistance variant resulted in the suspension of the active treatment or affected subsequent treatment choice in 9 (69%) out of 13 patients. However, the presence of a therapy sensitizing variant only led to consideration of a biomarker-directed therapy in 6 (10%) out of 61 patients. Conclusions: Overall, CGP of non-myeloid hematologic malignancies identified clinically significant variants in 72% of patients and resulted in a change in management in 22% of patients.

## 1. Introduction

The use of comprehensive genomic profiling (CGP) to inform diagnosis, prognosis, and therapeutic selection has become increasingly common in the management of myeloid and advanced solid malignancies [1]. Biomarker-driven precision medicine strategies have transformed the therapeutic landscape of these cancers and prompted the adaptation of novel clinical trial platforms [2,3]. However, the clinical utility of CGP in non-myeloid hematologic malignancies remains controversial, in part due to the limited number of biomarker-driven drugs available in this setting.

Although studies suggest that CGP frequently identifies potentially actionable variants in patients with non-myeloid hematologic malignancies, the definition of “potentially actionable” varies among studies, and the identified variants do not necessarily lead to changes in clinical practice [4,5,6,7]. For instance, one study found that 82% of patients with lymphoid malignancies who underwent CGP had a variant that could potentially guide therapy, but only 5% had a variant for which there was a United States Food and Drug Administration (FDA)-approved therapy for their diagnosis [5]. Another study found that CGP identified potentially actionable variants in 52% of patients with lymphoma who underwent CGP to assess for targetable alterations, but none of these patients received a targeted therapy based on the CGP results [6].

FoundationOne Heme is a commercially available DNA- and RNA-based CGP platform utilized for the sequencing of hematologic malignancies using formalin-fixed paraffin-embedded (FFPE) tissue samples, whole blood samples, or bone marrow aspirate samples [8]. When RNA sequencing is required, this CGP panel may also be used for sarcomas and other solid malignancies. The sequencing panel includes 406 DNA-sequenced genes and 265 RNA-sequenced genes, including known pathogenic gene rearrangements and fusions. In addition to clinical care, CGP platforms such as these have been adopted for use in large clinical trial efforts for acute myeloid leukemia (AML), including the Beat AML Master Trial which studied the use of CGP-directed care in untreated AML and demonstrated the feasibility of rapid personalized medicine in these populations [9].

In this study, we determined the rate of variants with potential diagnostic, prognostic, or therapeutic significance that were identified by CGP using FoundationOne Heme in patients with non-myeloid hematologic malignancies at our institution. To assess the clinical impact of these potentially actionable variants, we categorized the identified variants by evidence level and determined the rate at which variants found by CGP led to changes in clinical practice.

## 2. Materials and Methods

We identified patients who received care for non-myeloid hematologic malignancies at Duke University Medical Center (Durham, NC, USA) and who had CGP sent between October 2014 and November 2021 as part of routine clinical care. CGP was performed as part of routine clinical care by Foundation Medicine (Cambridge, MA, USA) using the FoundationOne Heme panel, which uses hybrid capture-based next-generation sequencing for the DNA sequencing of 406 genes and RNA sequencing of 265 genes [8]. Diagnoses were made by hematopathologists and clinical oncologists based on the 2016 World Health Organization classifications [10]. Patient characteristics, CGP results, and details on medical management as a result of CGP were abstracted from a retrospective review of medical records with the data cutoff date 4 February 2022. In addition, provider documentation of changes in medical care as a result of CGP data, including diagnostic and therapeutic management, or the consideration of a biomarker-driven clinical trial, were captured. Approval of this retrospective study was granted by the Duke University Institutional Review Board (protocol numbers Pro00102515 and Pro00101796) and included a waiver of informed consent.

CGP testing results at Duke University are stored and categorized in a secure and searchable clinical database called the Duke Molecular Registry of Tumors (MRT) as part of standard clinical operating protocol [11]. Patients who have undergone CGP testing are then reviewed at a weekly hematology-specialized multidisciplinary molecular tumor board (MTB). MTB meetings routinely analyze the significant findings within next-generation sequencing reports, review potential clinical applications among a group of disease experts, and subsequently share these discussion findings with the primary oncology team to assist in patient care.

In this study, variants of clinical significance were identified with the aid of the OncoKB precision oncology knowledge base (https://www.oncokb.org (accessed on 1 February 2022)) and classified by the level of evidence per the 2017 Joint Consensus Recommendations of the Association of Molecular Pathology, American Society of Clinical Oncology, and College of American Pathologists [12,13,14]. According to this classification, variants were categorized as having evidence level A (FDA-approved or included in professional guidelines), B (supported by expert consensus), C (investigational or FDA-approved for different tumor type), or D (supported only by preclinical data or case reports) based on information at the time of data abstraction. Variants with therapy sensitizing, diagnostic, or prognostic significance were classified as having evidence level A, B, or C. Therapy sensitizing variants with evidence level C were further subdivided into levels C1 (investigational) or C2 (FDA-approved for different tumor type). Variants associated with therapy resistance were classified as having evidence level A or D. Variants of clinical significance were defined as mutations with therapy sensitizing, diagnostic, or prognostic significance with evidence level A–C, as well as variants related to treatment resistance with evidence level A or D. The original literature supporting the evidence level of each variant is cited on the OncoKB website.

## 3. Results

### 3.1. Patients Undergoing Comprehensive Genomic Profiling

**Figure 1:**
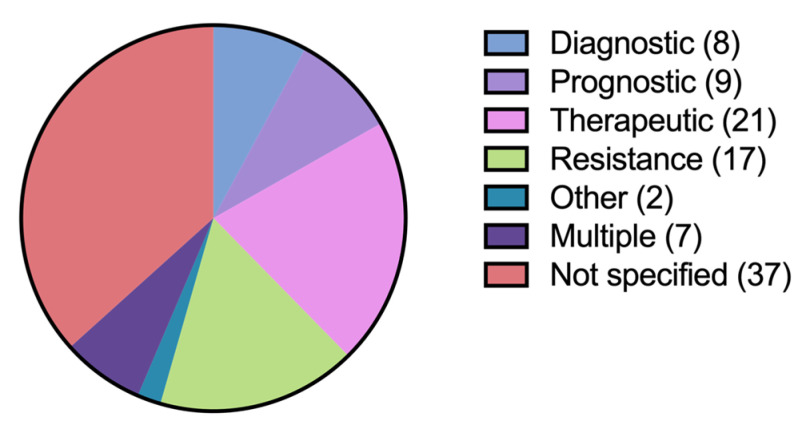
Clinician-stated justification for sending CGP. The number of patients falling into each category is depicted in parentheses in the legend. In some cases (*n* = 7), clinicians cited multiple reasons for sending CGP.

A total of 105 samples from 101 patients with non-myeloid hematologic malignancies were sent for comprehensive genomic profiling between 2014 and 2021 (Table 1). In four patients, CGP was sent twice. The median age of tested patients was 60 years. The most common diagnoses among tested patients were chronic lymphocytic leukemia (CLL), B-cell non-Hodgkin lymphoma (NHL), T-cell NHL, histiocytic neoplasms, and plasma cell neoplasms. Justification for sending CGP was often not specified in clinical documentation (Figure 1). When specified, the most common clinician-stated rationale for sending CGP were to direct therapy selection and to uncover resistance mutations.

The majority of specimens sent for CGP were bone marrow or peripheral blood (Table 2). Among the 105 samples used to perform CGP, 92 (88%) identified one or more pathogenic or likely pathogenic mutation. The most commonly altered genes were *TP53* (30 patients), *PCLO* (20 patients), and *NOTCH1* (20 patients). Notably, CGP data were only reported after 12 patients had already died, and within 30 days of death in an additional 11 patients (Table 1). Among 101 patients, 73 (72%) had at least one variant of clinical significance associated with diagnosis, prognosis, therapy sensitization, or therapy resistance.

### 3.2. Variants Associated with Therapy Sensitization or Therapy Resistance

**Figure 2:**
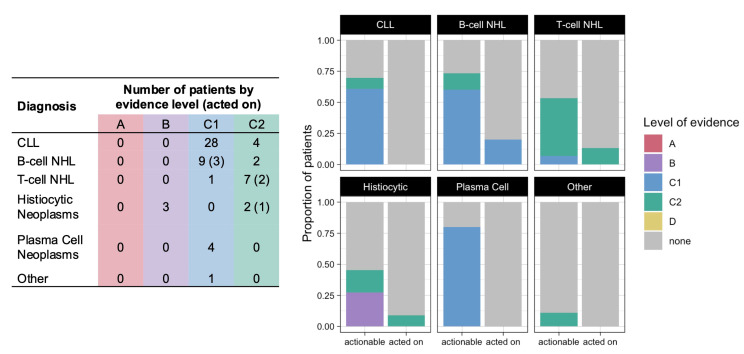
Number (table, (**left**)) and proportion (graph, (**right**)) of patients who had therapy sensitizing variants identified by CGP (actionable) and in whom these variants led to a biomarker-directed therapy being offered (acted on), color coded by level of evidence.

Among the 101 patients who underwent CGP, 61 patients (60%) had a therapy sensitizing variant, all with level of evidence B or C (Figure 2). The four variants with level B evidence were *KRAS* or *MAP2K1* mutations found in patients with histiocytic neoplasms, for which MEK inhibitors such as cobimetinib or trametinib could be utilized. The other variants had either level C1 evidence (investigational) in 42 patients or level C2 evidence (FDA-approved for a different tumor type) in 16 patients. Among the 61 patients with therapy sensitizing variants, 6 patients (10%) were offered a CGP-guided treatment regimen (Figure 2 and Table 3), of whom 3 patients actually received the therapy.

**Figure 3:**
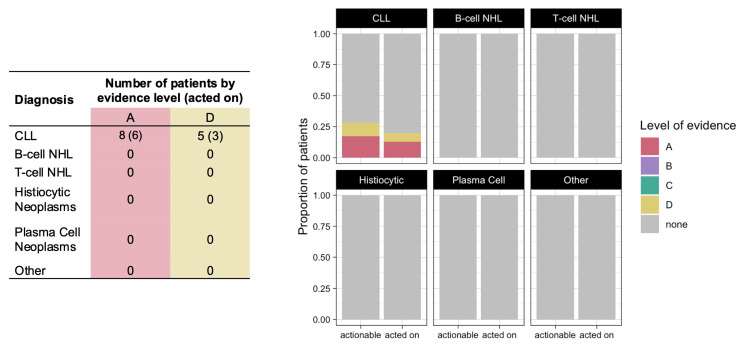
Number (table, (**left**)) and proportion (graph, (**right**)) of patients who had variants conferring therapy resistance identified by CGP (actionable) and in whom these variants resulted in suspension of the active treatment or affected subsequent treatment choice (acted on), color coded by level of evidence.

Variants conferring therapy resistance were identified in 13 patients (13%), all of whom had a diagnosis of CLL (Figure 3). The only identified resistance mutation with level A evidence was the *BTK* C481S mutation, which confers resistance to covalent Bruton tyrosine kinase (BTK) inhibitors. Variants with level D evidence included the *BTK* C481R mutation and *PCL2G* mutations, which confer resistance to BTK inhibitors, as well as the *BCL2* G101V mutation, which confers resistance to the B-cell lymphoma 2 (BCL2) inhibitor venetoclax [15,16]. Whereas therapy sensitizing variants rarely led to changes in clinical practice, the discovery of a resistance variant resulted in the suspension of the active treatment or guided subsequent treatment choice in 9 of the 13 patients (69%): 6 patients discontinued ibrutinib, 1 patient discontinued venetoclax, and 2 patients were treated with the non-covalent BTK inhibitor nemtabrutinib based on these CGP results. The absence of a resistance variant identified by CGP affected the treatment selection for another four patients.

### 3.3. Variants with Diagnostic, Prognostic, or Genetic Significance

**Figure 4:**
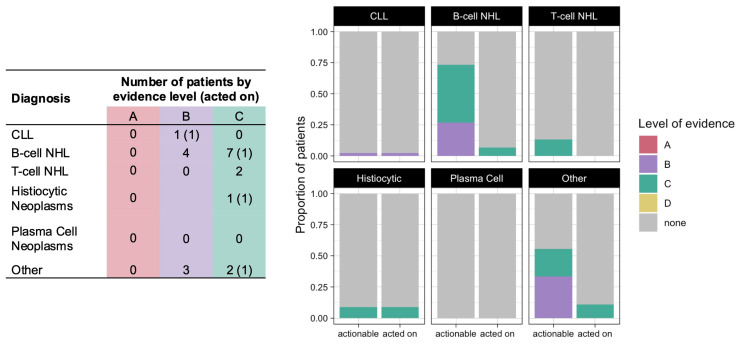
Number (table, (**left**)) and proportion (graph, (**right**)) of patients who had variants of diagnostic significance identified by CGP (actionable) and in whom these variants were used to clarify a diagnosis (acted on), color coded by level of evidence.

Among the 101 patients who underwent CGP, 20 patients (20%) had a variant of diagnostic significance, all with level of evidence B or C (Figure 4). However, CGP results were used to clarify the diagnosis in only 4 (20%) of these 20 patients: 1 patient with *SOCS1* truncation who was diagnosed with diffuse large B-cell lymphoma, 1 patient with *STAT3* Y640R mutation who was diagnosed with T-cell large granular lymphocytic leukemia, 1 patient with *ETV3–NCOA2* fusion who was diagnosed with indeterminate cell histiocytosis, and 1 patient with *MPL* and *CALR* mutations who carried a diagnosis of CLL but was additionally diagnosed with myelofibrosis based in part on these results.

**Figure 5:**
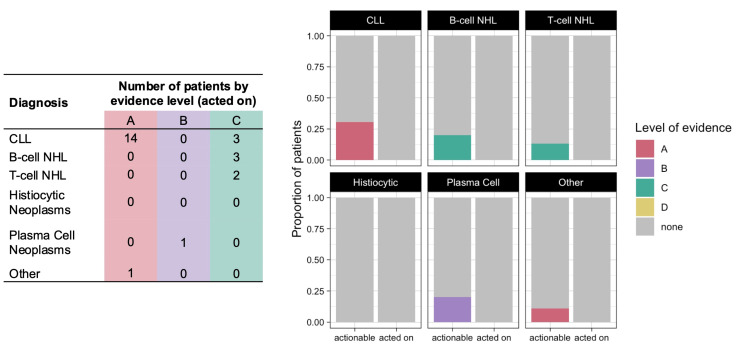
Number (table, (**left**)) and proportion (graph, (**right**)) of patients who had variants of prognostic significance identified by CGP (actionable) and in whom these variants led to change sin clinical practice (acted on), color coded by level of evidence.

Variants of prognostic significance were identified in 21 patients (21%), but none of the identified variants led to changes in clinical practice (Figure 5). CGP additionally resulted in genetic counseling consultation for three patients: one patient with CLL who was found to have a *BRCA2* mutation, one patient with CLL who was found to have an *MSH6* mutation, and one patient with marginal zone lymphoma who was found to have a *VHL* mutation.

## 4. Discussion

With advances in our understanding of the diagnostic, prognostic, and therapeutic significance of genomic alterations, as well as the increasing availability of biomarker-directed therapies, comprehensive genomic profiling plays an increasingly important role in clinical oncology, particularly for myeloid and solid malignancies. In non-myeloid hematologic malignancies, the clinical utility of CGP remains in question given that there are a limited number of variants supported by a high level of evidence and few FDA-approved biomarker-directed therapies.

In this retrospective analysis of patients with non-myeloid hematologic malignancies who underwent CGP in a large academic institution, we found that CGP frequently identified variants of potential clinical significance (72%), but only a fraction of identified variants led to changes in clinical practice (22%). Variants conferring therapy resistance were more likely to influence treatment decisions (69%) compared to therapy sensitizing variants (10%), while variants of diagnostic significance helped clarify a diagnosis in 20% of cases, and variants of prognostic significance did not change clinical practice in any cases. The frequency of practice-changing findings on CGP also varied depending on the type of malignancy. While patients with plasma cell neoplasms were identified as having prognostic and therapy sensitizing variants, none of these CGP findings led to changes in clinical practice. Notably, CGP was often sent late in the disease course, with CGP resulting within 30 days of death or after death in 23% of patients. This observation raises the possibility that CGP could change clinical practice in a larger proportion of patients if it is sent earlier in the clinical course.

Notably, variants conferring therapy resistance were only identified in patients with chronic lymphocytic leukemia, but not in any other diagnoses. Moreover, resistance mutations were only identified in a few genes (*BTK*, *PLC2G*, and *BCL2*), suggesting that a targeted mutation panel might be a more cost-effective approach that would achieve the same results as CGP when the goal is to identify variants that confer therapy resistance in CLL. A formal cost-effectiveness analysis in this setting may help guide future use of this technology. Furthermore, with the advancement of medical technology, both the cost and efficiency of genomic assays may improve dramatically leading to greater utilization of this product. The importance of establishing a multi-disciplinary molecular tumor board within a cancer center to facilitate interpretation and clinical management based on CGP results will likely increase as this technology continues to develop.

## 5. Conclusions

CGP impacts clinical decision making for a subset of patients with non-myeloid hematologic malignancies. However, the current status of the evidence for variants with significance for diagnosis, prognosis, or therapy may not support the universal use of CGP for non-myeloid hematologic malignancies, but suppinstead suggests that CGP should be individualized to the appropriate clinical setting, whereas other approaches such as targeted mutation panels may be more appropriate in certain circumstances. The utility of CGP will need to be re-evaluated periodically as the landscape of genomic biomarkers and biomarker-directed therapies for non-myeloid hematologic malignancies evolves.

## Figures and Tables

**Table 1:** Characteristics of patients who underwent CGP.

Characteristic	*N* = 101
Median age ^1^, years (range)	60 (13–86)
Male sex, *n* (%)	59 (58%)
Race, *n* (%)	
Caucasian	69 (68%)
African American	19 (19%)
Asian	4 (4%)
American Indian or Alaskan Native	
Other/unknown	
Diagnosis	
CLL	46 (46%)
B-cell NHL ^2^	15 (15%)
T-cell NHL ^3^	15 (15%)
Histiocytic neoplasms	11 (11%)
Plasma cell neoplasms	5 (5%)
Other ^4^	9 (9%)
CGP result to time of therapy change ^5^, days (range)	26.5 (1–105)
CGP result 1–30 days before death, *n* (%)	11 (11%)
CGP result after death, *n* (%)	12 (12%)
Deceased ^6^, *n* (%)	35 (35%)

^1^ At time of testing; ^2^ B-cell NHL diagnoses included diffuse large B-cell lymphoma (9 patients), Waldenström’s macroglobulinemia (3), marginal zone lymphoma (2), and mantle cell lymphoma (1); ^3^ T-cell NHL diagnoses included angioimmunoblastic T-cell lymphoma (6 patients), peripheral T-cell lymphoma not otherwise specified (3), adult T-cell leukemia/lymphoma (2), cutaneous T-cell lymphoma (1), hepatosplenic γδ-T-cell lymphoma (1), primary cutaneous cytotoxic epidermotropic γδ-T-cell lymphoma (1), and monomorphic epitheliotropic intestinal T-cell lymphoma (1); ^4^ other diagnoses included T-cell large granular lymphocytc leukemia (2 patients), T-cell acute lymphoblastic leukemia/lymphoma (2), B-cell acute lymphoblastic leukemia (1), T-cell prolymphocytic leukemia (1), B-cell prolymphocytic leukemia (1), hairy cell leukemia (1), and NK cell lymphoproliferative disorder (1); ^5^ among patients with change in therapy due to presence of therapy sensitizing or resistance mutation; ^6^ at time of data cutoff.

**Table 2:** Types of specimens sent for CGP.

Specimen	Number (%)
Bone marrow	38 (36%)
Peripheral blood	37 (35%)
Skin	7 (7%)
Lymph node	5 (5%)
Other ^1^	18 (17%)

^1^ Other specimen included mediastinum (*n* = 4), lung (2), soft tissue (2), bone (2), abdomen (1), pleura (1), spine (1), tonsil (1), breast (1), duodenum (1), head and neck (1), and liver (1).

**Table 3:** Therapy sensitizing variants identified by CGP that led to a biomarker-directed therapy being offered.

Diagnosis	CGP Result	Therapy	Best Response
Patients who received the therapy			
DLBCL (IVL)	*CD79B* mutation	ibrutinib	*
T-cell lymphoma (AITL)	*TET2* mutation	romidepsin	PR
T-cell lymphoma (cutaneous γδ)	*JAK2* fusion	ruxolitinib	PD
Patients who were offered but did not receive the therapy			
DLBCL (PMBCL)	*JAK2* mutation	ruxolitinib	
DLBCL (triple hit)	*NRAS* mutation	MEK inhibitor	
Histiocytic sarcoma	PD-L1 and PD-L2 amplification	nivolumab	

IVL, intravascular lymphoma; AITL, angioimmunoblastic T-cell lymphoma; PMBCL, primary mediastinal B-cell lymphoma; PR, partial response; PD, progressive disease. * Used as maintenance therapy.

## Data Availability

Please reach out to the corresponding author for data requests.
